# Endocrine dysfunctions in patients with mitochondrial diseases

**DOI:** 10.1186/1687-9856-2015-S1-O23

**Published:** 2015-04-28

**Authors:** Ja Hyang Cho, Ja Hye Kim, Jin-Ho Choi, Gu-Hwan Kim, Han-Wook Yoo

**Affiliations:** 1 Department of Pediatrics, Asan Medical Center Children’s Hospital, University of Ulsan College of Medicine, Seoul, Korea; 2 Medical Genetics Center, Asan Medical Center Children’s Hospital, University of Ulsan College of Medicine, Seoul, Korea

## Aims

Mitochondrial diseases are a heterogeneous group of genetic disorders that result from dysfunction of the respiratory chain. The endocrine disorders such as diabetes mellitus, hypoparathyroidism, hypothyroidism and growth hormone deficiency have been described in patients with mitochondrial DNA mutations. Among these, patients with Mitochondrial Encephalomyopathy, Lactic Acidosis, and Stroke-like episodes (MELAS) frequently present with mitochondrial diabetes. Thus, this study was performed to investigate endocrine dysfunctions in patients with mitochondrial diseases.

## Methods

A total of 106 patients (65 males and 41 females) diagnosed with mitochondrial diseases were included. Their etiologies were confirmed by clinical features and mitochondrial gene sequencing: Leber Hereditary Optic Neuropathy (LHON) (56 patients, 52.8%), MELAS (33 patients, 31.1%), Leigh disease (7 patients, 6.6%), Myoclonic Epilepsy with Ragged Red Fibers (MERRF) (7 patients, 6.6%), Pearson syndrome (2 patients, 0.02%) and Kearns-Sayre syndrome (KSS) (1 patient, 0.9%). Clinical, auxological and endocrinological parameters such as weight, height, body mass index (BMI), serum free T4, thyroid stimulating hormone (TSH), and glycosylated hemoglobin (HbA1c) were analyzed. Their medical records were reviewed retrospectively.

## Results

Endocrine dysfunctions were observed in 19 patients (19/33, 57.6%) with MELAS,1 patient with KSS and Pearson disease, respectively. In patients with MELAS, mean age at diagnosis was 15.2 ± 10.7 years (range, 8 month to 36 years). Their mean height, weight, and BMI were 139.0 ± 20.1 cm (- 2.54 SDS), 30.0 ± 11.6 kg (- 4.43 SDS) and 15.7 ± 3.4 kg/m^2^ (- 2.18 SDS), respectively. Mean HbA1c was 6.59 ± 1.81% in 24 patients with MELAS who showed hyperglycemia in routine serum chemical tests. Thyroid functions were normal in all patients. In mitochondrial gene analysis of MELAS, mt.3243 A>G was the most common (23/33 alleles, 69.7%), followed by mt.3316A>T (1 alleles), mt.13513G>A (1 allele), mt.8363G>A (2 allele), and mitochondrial DNA deletion (1 allele). One patient with KSS showed hypocalcemia with low parathyroid hormone level and stunted growth velocity. A Pearson syndrome patient manifested adrenal insufficiency. Endocrine dysfunctions were not found in the other mitochondrial diseases including LHON, Leigh disease, and MERRF.

## Conclusion

Endocrine dysfunctions and growth failure are associated with mitochondrial diseases, especially in patients with MELAS. However, their growth parameters can be affected by general condition, nutrition, and other associated factors. Mitochondrial endocrinopathy should be considered as a rare cause of endocrine dysfunction and growth failure when clinically suspected.

